# Longitudinal variation in *O*^6^-alkylguanine DNA-alkyltransferase activity in the human colon and rectum

**DOI:** 10.1038/sj.bjc.6600455

**Published:** 2002-07-02

**Authors:** N P Lees, K L Harrison, E Hill, C N Hall, G P Margison, A C Povey

**Affiliations:** Cancer Research UK, Carcinogenesis Group, Paterson Institute for Cancer Research, Manchester M20 9BX, UK; Department of General Surgery, Wythenshawe Hospital, Manchester M23 9LT, UK; School of Epidemiology and Health Sciences, University of Manchester, Oxford Road, Manchester M13 9PT, UK

**Keywords:** *O*^6^-alkylguanine DNA-alkyltransferase, MGMT, colon

## Abstract

In a systematic study of *O*^6^-alkylguanine DNA-alkyltransferase activity in the human colon and rectum, tumours were found to occur in regions of low activity. These results are consistent with the hypothesis that *O*^6^-alkylguanine DNA-alkyltransferase levels and alkylating agent exposure may be important determinants of large bowel tumorigenesis.

*British Journal of Cancer* (2002) **87**, 168–170. doi:10.1038/sj.bjc.6600455 www.bjcancer.com

© 2002 Cancer Research UK

## 

The DNA repair protein, *O*^6^-alkylguanine DNA-alkyltransferase (MGMT), provides protection against the carcinogenic, mutagenic and cytotoxic effects of alkylating agents (O'Connor *et al*, 2000). Thus, overexpression of MGMT in transgenic rodents protects against methylating agent induced GC→AT transitions in the *K-ras* oncogene and against colonic aberrant crypt formation (Zaidi *et al*, 1995). We have found an association between low levels of MGMT activity in adjacent normal mucosa and GC→AT transition mutations, but not transversion mutations, in *K-ras* of colorectal cancers (Jackson *et al*, 1997). Hypermethylation of the promoter region of the *O*^6^-alkylguanine DNA-alkyltransferase gene has similarly been associated with GC→AT transition mutations in *K-ras* of colorectal cancers (Esteller *et al*, 2000). Variations in MGMT activity may therefore determine susceptibility to mutagenic events due to endogenous and exogenous methylating agents in the human colon and rectum. MGMT activity in colorectal mucosa have been shown to vary widely between individuals (e.g. Gerson *et al*, 1995; Povey *et al*, 2000b) but the magnitude of intra-individual variation within the colon is largely unknown. To date, human studies have taken single biopsy samples to be representative of the normal mucosa adjacent to colorectal cancers without validation. Variations, if any, in MGMT activity within the normal colon may bias comparisons with tumour samples and between different studies. The aim of this study was to examine for the first time in a systematic manner, the topographical pattern of MGMT activity of normal mucosa around colorectal cancers and to determine the intra-individual variation in MGMT activity.

## MATERIALS AND METHODS

Mucosal biopsy samples were obtained from seven patients with tumours of the rectum, sigmoid colon and caecum. All but one patient was male and their age ranged from 48 to 83 years. Tumours were examined by a consultant histopathologist and the histological classification of the tumours is shown in Table 1

**Table 1:**
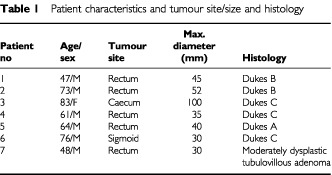
Patient characteristics and tumour site/size and histology

: the diameter of the tumours varied between 30 and 100 mm. Normal mucosa samples were harvested upstream (proximal) and downstream (distal) to the freshly resected tumours at 1 cm intervals for at least 5 cm, and at further intervals (in long specimens) until both resection margins were reached. Tumour samples (up to two) were taken along the same axis as the normal mucosa samples. All samples were collected by a single experienced colorectal surgeon (NP Lees). Care was taken to dissect the mucosa away from deep layers during harvesting of normal mucosal samples and to avoid areas of slough during harvesting of tumour samples. Samples were snap frozen and then stored at −80°C until assayed. All surgical specimens were examined by a consultant histopathologist to determine tumour stage and to exclude any background inflammatory bowel disease in the normal mucosa.

A MGMT activity assay was performed on each sample as previously described (Watson and Margison, 1999). Briefly, the tissue samples were thawed and 1 ml of ice cold buffer I (50 mM Tris-HCl, pH 8.3, 1 mM EDTA, 3 mM dithiothreitol) containing 0.5 mM leupeptin (Sigma) was added. The tissues were homogenised and sonicated, phenylmethylsulphonylfluoride (Sigma; 50 mM in 100% ethanol) was added to a final concentration of 0.5 mM, and the suspension centrifuged at 15 000 r.p.m. for 10 min at 4°C. The supernatant was removed and kept at 0°C. The DNA concentration of each extract was calculated, in duplicate, by measuring the fluorescence of known dilutions with Hoechst 33258 dye (1 μg ml^−1^) in TNE buffer (10 mM Tris base, 1 mM EDTA, 0.2 mM NaCl, pH 7.4) read on a Biolite fluorescent microtitre plate reader. Calf thymus DNA standards (Pharmacia biotech, ultrapure) of known DNA content were used for calibration.

The MGMT assay was performed three times for each extract, using different extract volumes and the amount of [^3^H]methyl groups transferred from substrate calf thymus DNA (Sigma) to protein, under conditions of excess substrate, was quantified by liquid scintillation counting. MGMT activity was expressed as fmole *O*^6^-methylguanine removed per μg DNA. All samples from the same patient were prepared and assayed simultaneously. The coefficient of variation within a triplicate assay was 7%; day to day variation in a control sample was 11%.

Differences in MGMT activity between normal and tumour samples were examined by ANOVA. The mean gradients of MGMT activity proximal and distal to the tumours were calculated and a one sample *t*-test, weighted according to the number of observations, used to test whether the mean gradients were significantly positive or negative. Gradients were assigned as negative if they increased towards the tumour (irrespective of proximal or distal direction).

## RESULTS AND DISCUSSION

All tumour samples (*n*=13) and 100 out of 102 normal mucosal samples contained detectable MGMT activity. MGMT activity varied between <0.25 (limit of detection) and 37.9 fmole μg^−1^ DNA. Mean MGMT activities (±s.d.), proximal and distal to the tumour, are shown in Table 2

**Table 2:**
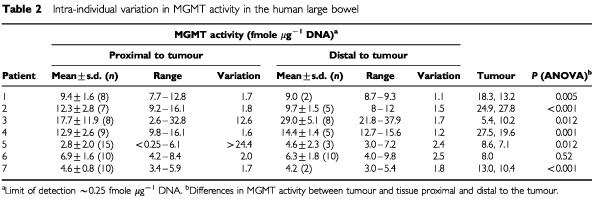
Intra-individual variation in MGMT activity in the human large bowel

. For five of the six tumours from the lower large bowel (sigmoid colon or rectum), MGMT activity was significantly higher in the tumour sample than in the surrounding tissue (Table 2) but there was little evidence of a field effect (i.e. tumour tissue exerting an effect on ATase activity in normal mucosa which was proportional to tumour proximity) within a limited region of the tumour. Such results are consistent with previously published studies (e.g. Povey *et al*, 2000b) showing increased MGMT activity in tumour samples: whether this increase reflects differences in cell type or is due to upregulation of gene expression or decreased exposure to alkylating agents (Povey *et al*, 2000a) and/or pseudosubstrates remains to be clarified.

There were no significant differences in mean MGMT activity distal or proximal to tumours of the rectum or sigmoid colon. For the caecal carcinoma (patient 3) activities distal to the tumour were higher than those proximal to the tumour (i.e. in the ileum: *P*=0.03) suggesting intertissue differences in MGMT activity. Proximal to all tumours the inter individual variation was ∼6.3-fold whereas the intraindividual variation varied between 1.6 and >24. Distal to tumours, the inter individual variation was ∼6.9-fold whereas the intraindividual variation varied between 1.1 and 2.5 (Table 2).

**Figure 1:**
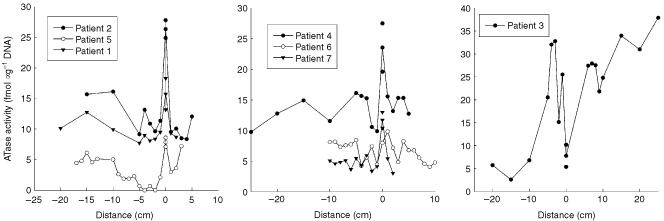
MGMT activity (fmoles μg^−1^ DNA) upstream (proximal) and downstream (distal) to colorectal tumours. The tumour is located at 0 cm: negative and positive distances are respectively upstream (proximal) and downstream (distal) from the tumour. As two samples were taken from each tumour, both the individual activities and the mean MGMT activity is shown.

A consistent topographical pattern of MGMT activity in normal mucosa associated with colorectal cancers was observed with tumours occurring in regions of low MGMT activity. There was a modest but significant fall in MGMT activity, unrelated to tumour subsite or stage, upstream of left sided tumours (Figure 1

). The gradient, proximal to the tumour, ranged from −0.07 to 0.44 fmoles μg^−1^ DNA per cm (Table 3

**Table 3:**
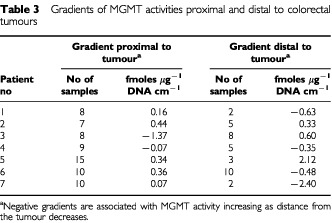
Gradients of MGMT activities proximal and distal to colorectal tumours

). The mean gradient was 0.22 fmole μg^−1^ DNA per cm (95% CI=0.03–0.42: *P*=0.02). Over a 10 cm length of tissue, this would correspond to between a 10–80% drop in MGMT activity (based upon mean values shown in Table 2). Distally, MGMT activity fell as the distance from the tumour increased with a mean gradient of −0.17 fmoles μg^−1^ DNA (95% CI=−1.35–1.01) but the small distal resection margins resulted in a limited number of assayable samples. Activity adjacent to the caecal tumour was lowest in the ileum, and increased with distance distal to the cancer (Figure 1: patient 3). The gradient was −1.37 fmole μg^−1^ DNA proximally and 0.60 fmole μg^−1^ DNA distally.

The gradient in activity along the colon may reflect a number of different mechanisms including longitudinal changes in gene expression or variable exposure to alkylating agents (or pseudosubstrates). If tumours form in areas of low MGMT activity, this would suggest that MGMT activity is a factor predetermining susceptibility to colorectal cancer, a hypothesis consistent with our understanding of the known actions of MGMT in providing protection against the mutagenic and carcinogenic effects of alkylating agents (O'Connor *et al*, 2000). If however, the tumour site is determined by increased exposure to alkylating agents, the MGMT gradient may simply reflect this exposure and is not a factor determining susceptibility. Consistent with this latter hypothesis we have previously reported that *O*^6^-methylguanine levels were higher in the sigmoid colon and rectum than in the caecum (Povey *et al*, 2000a). It remains to be established whether this gradient is a reflection of recent exposure to alkylating agents or perhaps low MGMT activity present earlier at the time of tumour initiation.
